# Youth mental health and/or addiction concerns and service needs during the COVID-19 pandemic: a qualitative exploration of caregiver experiences and perspectives

**DOI:** 10.1186/s13034-022-00471-0

**Published:** 2022-05-10

**Authors:** Roula Markoulakis, Andreina Da Silva, Sugy Kodeeswaran, Anthony Levitt

**Affiliations:** 1 Sunnybrook Research Institute, 1929 Bayview Avenue, Toronto, ON M4G 3E8 Canada; 2 Department of Occupational Science and Occupational Therapy, University of Toronto, 500 University Avenue, Toronto, ON M5G 1V7 Canada; 3 Family Navigation Project, Sunnybrook Health Sciences Centre, 1929 Bayview Avenue, Toronto, ON M4G 3E8 Canada; 4 Hurvitz Brain Sciences Program, Sunnybrook Health Sciences Centre, 2075 Bayview Avenue, Toronto, ON M4N 3M5 Canada; 5 Department of Psychiatry, University of Toronto, 250 College Street, Toronto, ON M5T 1R8 Canada

**Keywords:** Youth mental health and addictions, Caregivers, COVID-19, Pandemic, Virtual care, Mental health services

## Abstract

The COVID-19 pandemic has undoubtedly had a significant impact on youth mental health and/or addiction concerns and exacerbated pre-existing gaps in access to mental health and/or addiction care. Caregivers can support their youth in seeking and participating in care, however, their experiences in doing so in the pandemic and their perspectives of their youth’s care needs are not well-understood. A descriptive qualitative study was conducted to better understand youth’s and caregivers’ experiences accessing care during the pandemic, from the caregivers’ standpoint. Participants completed semi-structured qualitative interviews that focused on experiences seeking and accessing mental health and/or addiction services, with specific questions regarding their experiences accessing services during the pandemic. A total of 46 interviews were included in the thematic analysis of the data. Study findings highlight the impacts of the pandemic on everyday life, on youth mental health and/or addiction needs, on caregiver’s experiences seeking and accessing services for their youth, and on service access, including perceptions of virtual care modalities. The discussion highlights the importance of focusing on factors that enable youth’s participation in care, to ensure accessibility of appropriate and timely care that meets youth’s and families’ needs. Also highlighted is the frustration and despair experienced by caregivers seeking critical mental health and addictions supports for their youth during the pandemic, as well as the sense of isolation and of being left behind by the system. Identifying caregiver perspectives of youth mental health and addiction needs and service access during the COVID-19 pandemic provided important insights that can help inform approaches to youth mental health and addictions care during the pandemic and beyond.

**Supplementary Information:**

The online version contains supplementary material available at 10.1186/s13034-022-00471-0.

## Introduction

The COVID-19 pandemic has created profound psychological distress in society. While mental health and addictions (MHA) issues have historically been estimated to affect one in five Canadian youth at any given time [1], the pandemic has likely increased that proportion. Recent evidence suggests Canadians have been experiencing worsening mental health as a result of the pandemic [2], and youth and caregivers may be particularly vulnerable [3–8]. Youth have indicated considerable negative impacts to their mental health during the pandemic [4, 5]. While substance use among youth was generally found to decrease, problematic levels of use were identified in those who were engaging in substance use [5]. Increases in youth MHA concerns have also been identified as a result of stress [7], fear of COVID-19 itself [3], social isolation, and peer pressure and judgement related to pandemic behaviours [9]. These concerns may be linked to the disruption of youth’s daily routines, including interruption to school routines and decreased interactions with peers [10], and underline the need for appropriate and available MHA supports for youth.

As youth report perceived changes to their mental health and show increased rates of MHA concerns, families, and specifically caregivers, may identify their youth’s MHA needs, aid their youth in coping and dealing with their concerns, and become actively involved in their care and treatment plans [10, 11]. Caregiver strain is also likely heightened, due in part to employment/financial issues and changes in caregiving responsibilities [6]. Caregivers have identified a pressing need for youth mental health supports as well as caregiver-specific strategies and family targeted interventions to aid caregiver’s stress and emotional well-being as they tend to their youth’s needs during the pandemic [6]. These identified needs highlight the importance for both youth and caregivers of having available and accessible MHA services during the pandemic.

In addition to the potential for negative MHA effects, the pandemic has exacerbated pre-existing gaps in care and led to disruptions in services for those already connected (e.g., closures, support group cancellations, moves to phone or online services), or difficulties accessing MHA supports in the first place [12–14]. Many MHA services rapidly adapted in response to pandemic limitations, providing virtual supports, changing operating hours and intake rules, or implementing physical distancing [13–15]. While it is clear that ample efforts have been made to increase the capacity of existing MHA services to support those who are struggling during the pandemic, gaps and barriers are present nonetheless. Providers have noted difficulties in making service referrals, due to a lack of available services, as well as increased workload on staff working in mental health services [16]. Providers have also expressed doubts regarding their ability to provide adequate assessments and form rapport with service users virtually [16]. Virtual care barriers for patients include access to technology, computer literacy, patient resistance or mistrust, language, and privacy [17, 18]. These shifts have resulted in disruptions to the management of pre-existing MHA concerns and difficulties accessing needed MHA care among youth [19, 20].

While caregivers can play a significant role in motivating and facilitating the care plan of youth experiencing MHA concerns, the impacts of the COVID-19 pandemic on youth MHA service needs and experiences, from the perspectives of caregivers who take on this role, is not well known. Developing such understanding may lead to system improvements that will ensure that youth MHA needs can be better addressed during the COVID-19 pandemic and beyond. Thus, the purpose of the current study was to explore caregivers’ perspectives and experiences in seeking and accessing youth MHA care during the COVID-19 pandemic.

## Methods

### Design

A descriptive qualitative approach was utilized to explore and lend a multifaceted view of caregivers’ experience looking for and accessing MHA services for their youth and/or themselves as caregivers during the pandemic [21]. This data was collected as part of a larger ongoing clinical trial (NCT03765281) focused on testing interventions that support access to and transitions through youth MHA care [22]. The Sunnybrook Health Sciences Centre Research Ethics Board approved the larger ongoing study as well as the focused amendment to explore caregiver experiences during the pandemic.

### Setting and study population

This study was conducted by a research team based in an academic health sciences centre in Toronto, Ontario, Canada. Early in the pandemic and during the time the study interviews were conducted, Toronto had experienced the greatest number of lockdown days in the world [23], along with related stay-at-home orders, school closures/virtual learning, and MHA service changes. Participants were caregivers of youth ages 13–26 with mental health and/or addictions (MHA) concerns who were 1. residing in the Greater Toronto Area and 2. interested in assistance with identifying and accessing appropriate MHA supports for their youth. Participants were recruited through social media advertisements (i.e., Facebook, Instagram). See Table 1 for participant characteristics.

**Table 1:** Participant background characteristics

	n (%)/M (SD)
Age (years)—caregiver (n = 46)	51.52 (5.31)
Gender—caregiver	
Female	46 (100%)
Age (years)—youth (n = 46)	19.18 (3.40)
Gender—youth	
Female	23 (50%)
Male	20 (43.5%)
Transgender	2 (4.3%)
Other	1 (2.2%)
Sexual orientation—youth	
Heterosexual	31 (67.4%)
Bisexual	4 (8.7%)
Gay/lesbian	3 (6.5%)
Other	2 (4.3%)
Preferred not to answer	1 (2.2%)
Unknown	5 (10.9%)
Living situation—youth	
Lived in the family home	41 (89.1%)
Away at school	1 (2.2%)
Lived independently	2 (4.3%)
At treatment centre	1 (2.2%)
Preferred not to answer	1 (2.2%)
MHA concerns—youth	
Mental health concerns only	30 (65.2%)
Addiction concerns only	2 (4.3%)
Concurrent concerns	14 (30.4%)

### Data collection

Participants completed semi-structured qualitative interviews by phone with the study research assistant (AD), lasting from twenty four minutes to one hour. Trial recruitment was paused per institutional mandate at the onset of the pandemic in March, 2020, and was relaunched in June, 2020. Interviews focused on participants experiences seeking and accessing MHA services, with specific questions regarding their experiences during the pandemic (i.e., “Has the pandemic affected your experiences in looking for services?”) and accessing services during the pandemic (i.e., “Has the pandemic affected your experiences in accessing/participating in services?). The pandemic-related questions were added upon relaunch of the trial. The interviews analysed as part of the current study were conducted at the baseline time-point of participants’ involvement in the aforementioned larger trial, to understand their experiences prior to receiving any intervention offered through the larger trial. As such, the interviews included in this current study were conducted between June 15, 2020 and February 11, 2021. All interviews were recorded and transcribed verbatim. A total of 64 interviews were conducted during this time, and 46 were included in analysis (transcript selection described below).

### Data analysis

As outlined by Braun and Clarke [24], a thematic analysis was conducted consisting of six phases. Two researchers from the team participated in all phases of the analysis (RM and AD). In the first phase of analysis, the two researchers read the transcriptions and gained familiarity with interview content. They also selected the interviews that contained pandemic-related segments to be analyzed. Given that not all participants had information to share in regards to their experiences during the pandemic, 18 of 64 interviews were excluded from the current study on this basis. After selecting the interviews, the two researchers developed appropriate codes from the data, using MAXQDA 2020. These codes were discussed to develop a preliminary codebook. This codebook was used to guide coding of the remaining interviews, and was regularly updated to reflect any new codes, deleted codes, or modification of codes and their meanings. These updates were agreed-upon through regular meetings between the two researchers. All codebook changes were tracked to maintain an audit trail and allow for previous interviews to be updated reflecting new, deleted, and updated codes. In the third phase, preliminary themes and sub-themes were developed. The researchers also kept analytic memos to track interpretations arising during coding that could later inform theme development [25]. Through the use of the memos, and through discussion between the two researchers, the preliminary themes and sub-themes were developed and codes were grouped accordingly in a manner that reflected patterns in the data. In phase four, the themes were refined and reviewed once again through discussion, and in phase five the themes were given appropriate names and were defined to provide an analytical explanation of each theme. The final phase involved writing up the findings of this analysis.

### Findings

Results of thematic analysis identified seven key themes that encompassed caregivers’ experiences in seeking MHA services for their youth during the pandemic. These included *changes to everyday life, MHA considerations during the pandemic, difficulties accessing MHA services, availability of MHA services, positive aspects of virtual care, negative aspects of virtual care, and caregiver involvement in help-seeking and access.* Subthemes within these themes are indicated in italics in each respective section. Themes and subthemes are summarized in Table 2.

**Table 2:** Study themes and subthemes

Themes	Subthemes
Changes to everyday life	Changes in daily routines Increased technology use Impact of changes in schooling Changes in social contacts
MHA considerations during the pandemic	MHA effects of pandemic Unclear MHA effects of pandemic MHA awareness
Difficulties accessing MHA services	Negative perceptions of service availability Extensive wait times Communication difficulties Interruptions to care Suboptimal support
Availability of MHA services	Positive impacts to youth service access Positive experiences of service availability Facilitation through school
Positive aspects of virtual care	Benefit of virtual format Rapport in virtual care Youth comfort with virtual care
Negative aspects of virtual care	Challenges with virtual care Difficulty with rapport in virtual care Discomfort with virtual care Comfort with in-person care
Caregiver involvement in help-seeking and access	Role in help-seeking and access Experiences in help-seeking and access

### Changes to everyday life

Participants described numerous negative and positive changes in various areas of their and their youth’s daily lives as a result of the COVID-19 pandemic. For example, both youth and caregivers experienced *changes in daily routines*, and caregivers connected these changes to their youth’s mental health needs: “being off for so many months, going back to a regular routine was probably stressful. Or at least a trigger…I know if she doesn’t have proper regular sleep patterns that her anxiety heightens as well” (P093). *Increased technology use* was also apparent and related to changes in routine, with caregivers emphasizing the impact on their youth: “She’s also completely lethargic, she’s completely unmotivated. She’s completely using her phone and technology as a distraction” (P152). Caregivers also described the notable *impact of changes in schooling* early in the pandemic, and the effects these changes had on their youth’s mental health. One caregiver stated: “I think a lot of struggles that [youth] had were brought on by the isolation with the pandemic and the fact that he couldn’t go to school and he couldn’t be around his friends” (P084). While school closures were noted for their negative impact on youth mental health, eventual school re-openings were also recognized for their perceived benefits: “Now I think it has greatly benefitted him that he’s returned to school so he’s got some level of social interaction that he didn’t have from March through to September” (P105). In contrast, youth who had seen improvements in their mental health during school closures experienced increased MHA difficulties upon return to in-person learning; as one caregiver stated: “if anything, [youth] started getting more anxious when she went back to school” (P093). Finally, *changes in social contacts* were also described. Outside social engagements and interactions became limited while time spent with family increased. Caregivers noted that “they need to socialize right now and this pandemic is really putting a lot of stress on the youth today” (P122), which sometimes led to strain in family relationships (see Additional file 1: Table S1, #1).

### MHA considerations during the pandemic

Caregivers consistently described important aspects of their own and their youth’s MHA concerns that were brought to the surface or altered during the pandemic. These *MHA effects of the pandemic* were often described as resulting from the above-mentioned changes to previously known ways of life:

“Not only was he not getting supports but he was also at home because school was shut down, meaning he was more isolated and less involved with friends and [had] less support, and I think that had a big toll…I think that impacted his anxiety levels. COVID has wreaked havoc on a lot of people’s anxiety.” (P105; see also Additional file 1: Table S1, #2)

Caregivers also attributed these changing MHA needs to lockdowns: “During the pandemic lockdown seems to be when it hit her the most…she had some suicidal ideation, high anxiety, she didn’t want to be alive anymore” (P121) along with attributions to the pandemic more generally: “With COVID she’s getting sad again. She doesn’t want to hang out with her friends, she’s hanging out in her bed all the time” (P142). Some caregivers also indicated that increased time together had made them more aware of troubling youth behaviours during the pandemic: “He’s at home alone most of the time with lockdown and he smokes [cannabis], so it’s not a social thing…so that’s what I wasn’t happy about” (P157). Time together at home also helped caregivers “just monitor a little better” (P097), lending assurance that the youth was not engaging in concerning behaviours and helping caregivers feel better able to support the youth. One caregiver shared: “We started to make some headway. The pandemic started, she was at home, less able to go out, less able to do things that were going to put her in jeopardy, so we were in a better situation” (P152; see also Additional file 1: Table S1, #3). As such, caregivers also indicated positive impacts on their youth’s MHA concerns, since situations that previously triggered depression, anxiety, or substance use were no longer prevalent:

“We’re in a much better spot right now…the fact that she’s at home and schooling online creates a lot less anxiety for her because she doesn’t have to face people…That stress and that volatility has been eliminated…so she’s actually much calmer” (P152; see also Additional file 1: Table S1, #4)

In other cases, caregivers were not certain that there had been any change in their youth’s MHA concerns, indicating *unclear MHA effects of the pandemic*. As one caregiver stated: “Is it because of the stress of COVID? Is it because of the therapy? I never really know, I feel kind of lost with this” (P040). Another caregiver also noted that it was difficult to be certain whether substance use behaviours had changed: “I’m not sure, I can’t be 100% sure but I have a feeling his usage went down a little bit.” (P157) Moreover, caregivers also emphasized the importance of being careful not to misattribute youth behaviours to current circumstances and overlook youth’s MHA needs, stating:

“[Therapist] kept saying ‘When he’s out with friends’ and I said ‘I don’t know what you’re talking about he doesn’t have any friends. He never leaves the house.’ She had no idea about that so it just shows that she missed that huge part of why he’s unhappy, she assumed he’s not leaving the house because of COVID” (P042).

Regardless of whether there had been changes in MHA concerns, caregivers noted *MHA awareness* as an important consideration during the pandemic. Many described an apparent reduction in MHA stigma during the pandemic:

“There is an awareness that kids are struggling…if anything I feel like there’s more conversation and support around kids right now than prior to the pandemic. I think for kids themselves it seems to be less stigmatized, I think the pandemic and the talk around it has de-stigmatized mental health issues for kids” (P137).

In contrast, some caregivers expressed concern over the perceived possibility that the pervasive discussions and attention paid to MHA in traditional and social media could lead more youth to believe they are experiencing MHA concerns: “It’s very much in the forefront right now with COVID, it’s on the news, it’s on social media, and it’s a conversation within all of their peer groups. I’m worried that has a contagion effect that’s going to impact them” (P118). Finally, caregivers shared that youth had voiced worry about the potential of the pandemic to negatively affect the mental health or physical health of their peers or loved ones: “She said ‘you know mom? I’m a bit of an introvert so I’m not too worried about me in this COVID time but some of my friends are extroverts and I’m worried about them’” (P053). These concerns for loved ones, in turn, could contribute to youth distress (see Additional file 1: Table S1, #5).

### Difficulties accessing MHA services

Caregivers had experienced considerable difficulties and barriers trying to find or access MHA care for their youth during the pandemic. As such, there were *negative perceptions of service availability*. Those trying to find or access services for the first time, or different than those with which they had been previously connected, indicated that with all of the shifts in MHA services offered that occurred during the pandemic, they were uncertain what services were available to them: “We never got on [the list] to be able to go there, plus COVID came in and I don’t know if you can call for that service right now” (P145). Caregivers also indicated that “mostly it’s been a lot harder to get services during the COVID situation” (P105), and that services seemed overburdened due to the pandemic in that services were unable to assist the volume of people seeking help:

“They closed programs…they’ve been relocated to [hospital] so the waiting list was even longer because now they’re coming from all over…All because of COVID and there’s more kids suffering and these kids are getting sicker. Also we were told that had these been normal times our daughter would’ve been admitted to a bed but they had to [change] the standards, the kids had to be even sicker to get help” (P114; see also Additional file 1: Table S1, #6).

In other cases, caregivers experienced cancellation of programs they had waited for or participated in prior to the pandemic. One caregiver stated: “The program that she was doing…it was from last September to April and because of COVID, in March it stopped” (P144), leading to frustration and a sense of wasted time:

“After that appointment with the psychiatrist at [hospital] they said he would be put on the waiting list for…an outpatient clinical program and they would contact us when the program started…That whole time he was seen once by the psychiatrist who made a recommendation to put him on [medication] but at no point did he have follow up counselling...It was ‘okay we’ll get you into this program’ but he waited and waited and when finally they were going to schedule him in they said they’re suspending the program” (P105; see also Additional file 1: Table S1, #7).

Caregivers also shared considerable difficulties experienced by their youth in relation to access needs, indicating that location, safety, and eligibility were of particular concern:

“If my son is living in a shelter and he has a follow-up appointment for the clinic at the hospital, it is uncommon of him to show up. [They’re] assuming he has transportation while we live way north of the hospital and it’s an hour and a half plus drive, and during COVID we don’t want him on public transport and if he’s not in communication with us cause he’s in the shelter it just—So transportation was an issue, communication was a huge issue, even my son’s willingness to participate was an issue, the all or nothing kind of approach like ‘we have no services to offer you unless you meet our criteria’ so that also put barriers in place for us that were very hard to overcome” (P067).

Service affordability was also a significant accessibility concern due to constraints on household finances. One caregiver shared: “my husband is still not working from the pandemic, it’s not something we can pay out of pocket for**.** So we’re limited to free services, which makes a difference” (P121). Together, these factors created a general perception that “because of COVID and everything there was really nothing available” (P084) in terms of MHA services. In some situations, caregivers reported that available support was insufficient: “She’s seeing a psychologist for therapy the last four weeks, every Sunday night, but she needs a little bit more than that.” (P089). Caregivers also noted *extensive wait times* for services. Waits for support were perceived to have increased in that “[the pandemic] also [creates] an extended waitlist because of the amount of people now seeking assistance” (P146; see also Additional file 1: Table S1, #8). Related to this, participants shared that they had experienced *communication difficulties* with providers. Caregivers noted “lag time with responding because of COVID…there’s a lot of leaving messages and calling back” (P136) and had trouble reaching service providers or did not hear back from providers for long periods of time:

“[Youth] went and saw a support worker… And she got along very well with her and we were really hoping she’d be able to talk to her more frequently, but then I don’t know what happened, COVID, everything just sort of stopped. Then when I did contact her I don’t know whether they weren’t working or she had quit because I hadn’t heard from her since” (P082; see also Additional file 1: Table S1, #9).

Those who had previously been connected with care for their youth described *interruptions to care* that had taken place during the pandemic, although in some instances, caregivers were uncertain if the interruptions were due to the pandemic. One caregiver stated: “[it’s] very hard to know if it was the pandemic or just general lack of services in that area because they’re both the case” (P125). Others felt strongly that interruptions were a result of the pandemic:

“I was very frustrated because it’s not my fault, we had faxed everything in so I don’t know how they got lost. Then the doctor I tried to contact to see if he would fill out the forms again, he’s now only doing emergency patients so we have to wait I guess until COVID is more under control” (P119; see also Additional file 1: Table S1, #10).

These interruptions sometimes resulted in caregivers attempting to “catch up” on appointments for their youth once restrictions lifted: “after things started to open we were so busy trying to get all our [cancelled appointments]…it’s been non-stop trying to catch up, especially now because I want to make sure everything gets done before potentially being shut down again” (P071). In cases where caregivers were ultimately able to access care for their youth, many described *suboptimal support*. Caregivers often felt compelled to accept the option that became available to them, stating “we’ll take what we can get” (P053), because “there are lots of people looking for help especially in the times of pandemic, so I can’t expect to get the exact match” (P157) indicating they would accept available options whether or not they felt as though it would be the most appropriately helpful care for their youth. Existing pandemic protocols at services were often viewed as necessary but as an element that prevented full comfort in or effectiveness of the care experience:

“Let’s say the groups starts at ten she would leave her home at six-thirty in the morning or seven to get there because that’s how she feels comfortable doing it, otherwise she gets too stressed out. And she was good with that because she would go there, get herself a [coffee shop] drink, and sit down in the waiting area and read a book and wait for the group, even if it was three hours long. It was her outing and she loved to do that until they cut that all off so when COVID was beginning they got very upset with her because she can’t come until the right time and that stressed her right out and she didn’t even want to go.” (P119; see also Additional file 1: Table S1, #11).

Caregivers shared that these experiences of suboptimal care resulted in unmet needs in their youth, which led families to rely on crisis supports:

“It’s been a long process but really complicated by COVID. My son was in a shelter. He was moving from shelter to shelter and he broke quarantine and they said “sorry you can’t come back in” and he had nowhere to go, and I couldn’t get any services, nothing, not even a place for him to stay. So he would get into a crisis situation…While we were in crisis we couldn’t access anything except the police…It was a very difficult time and even throughout the pandemic we have not received any services because they’ve been shut down because of COVID. It’s like a huge silence, there’s nothing. It’s very isolating, just feeling like you have to deal with this on your own cause nobody’s going to help you” (P067).

### Availability of MHA services

In some instances, caregivers shared experiences of accessing services during the pandemic without difficulty, describing *positive impacts to youth service access*, as a result of the pandemic. One caregiver stated that “if anything, I would say it’s almost been better than previous” (P137). For some, this meant that access to service was increased or there were better experiences accessing services during the pandemic. Another caregiver who was pleased with the timely appointment received by her youth shared: “COVID did not create a barrier and who knows it might have facilitated things because there’s possibly more of an awareness that mental health issues might be difficult in COVID so maybe there were more resources” (P039). *Positive experiences of service availability* were also described. One caregiver indicated that services were not as busy and therefore available to support their youth: “[youth] got a text from the psychiatrist two days later, I was like ‘wow I thought we’d be waiting months,’ I was shocked. But I think with this COVID all the specialists aren’t that busy…I was very happy” (P053). The flexibility offered by services being available in different formats, whether in-person or virtual, also resulted in service access that might otherwise not have been possible. One caregiver shared: “Because of COVID now they’re not meeting in the office, they meet outside the office at the picnic table outside…and she sees him once a week” (P031), and another caregiver stated: “The online stuff has been a life saver in this COVID situation for sure. The technology’s really helped a lot of people” (P053). Caregivers also indicated that providers had been able to take time to develop rapport with the youth, and these efforts were viewed positively: “He was flexible and he listened and he was funny. So there was more of a relationship. He was very human about things” (P036; see also Additional file 1: Table S1, #12).

Finally, *facilitation through school*, where the youth’s school was involved in facilitating access to services through direct school-based services or community-based services, was viewed favourably. Schools also provided care remotely, which was helpful to families during lockdowns or times of difficulty connecting with other supports (see Additional file 1: Table S1, #13). For some, supports provided through the school were all that was available to the youth. One caregiver indicated: “In terms of actual supports right now the only thing we have are the child and youth workers through the school” (P105; see also Additional file 1: Table S1, #14). Thus, caregivers were grateful for school involvement in the youth’s circle of care.

### Positive aspects of virtual care

Participants identified numerous positive experiences receiving MHA care in virtual formats, for both youth and caregivers. Caregivers described numerous *benefits of the virtual format*, such as choice in environment as a result of not attending appointments at a fixed location. Especially as a result of the pandemic, caregivers “thought it was great they had the opportunity to do that because obviously you can’t actually go to the [service location]” (P144), and as such, virtual care was also touted for time-saving benefits and limiting disruptions to everyday life:

“He can control his own environment while interacting with someone else…There’s a whole bunch of improvements in moving to virtual because you don’t have the anxiety-producing situation of ‘I have a doctor’s appointment today so I have to get pulled out of a physical classroom and go to a physical place and feel anxiety as I wait in that physical place feeling like there’s something wrong with me’” (P137; see also Additional file 1: Table S1, #15).

Participants also described their own or their youth’s disinclination for in-person services, due to safety concerns:

“Because of her autoimmune diseases, she doesn’t want to go out, even if there was something she could do a drop-in to. She doesn’t want to go in a subway, she doesn’t want to go in [a taxi], she doesn’t want to go somewhere she doesn’t have to go” (P040; see also Additional file 1: Table S1, #16).

Caregivers were also especially supportive of virtual care in instances when they felt service providers were striving to develop *rapport in virtual care.* This was evidenced when providers made effort to develop rapport with the youth as well as with the family, even though it could be more challenging in virtual settings:

“We think they have a good rapport, we think he’s got exactly the type of knowledge that [youth] needs. It’s all been done virtually of course, on the last meeting he got [youth] to invite my husband to join. There’s a lot of tension between all of us…So [provider] seems very knowledgeable. It does seem to be working” (P125).

Finally, caregivers suggested that the *youth’s comfort with virtual care* was an important factor in positive experiences. Care experiences were perceived to be more likely to proceed favourably if youth were open to accessing virtual care, as in one instance shared by a caregiver: “I didn’t know that [youth] could text [service] as well and that was a huge relief for [youth] because she didn’t have to talk to somebody” (P121), or even comfortable with the virtual format due to adaptation to virtual communication over the course of the pandemic or general familiarity with virtual formats prior to the pandemic. One caregiver shared that the youth’s opinion of virtual care had changed: “when everything transitioned to virtual he had a huge difficulty, he just couldn’t follow, couldn’t pay attention to it. But now he expresses that he actually thinks the virtual format is working better than in person” (P137). Despite this, one caregiver noted that although youth were “especially comfortable nowadays with the virtual format, everybody misses face to face though” (P153).

### Negative aspects of virtual care

Caregivers described difficulties with participation in virtual care and the perceived benefits of in-person care. Caregivers emphasized *challenges with virtual care*, particularly in cases where caregivers felt that “virtual group appointments [have] a total lack of engagement on [youth’s] part. They’re not useful at all…He logs onto the call and then just plays video games right through it” (P042), suggesting that youth were disengaged in virtual format:

“If you’re in [an in-person] group format you have to be more present, they can coax more out of you…whereas he was sitting in his bedroom, if he didn’t want to talk he didn’t talk. I would’ve liked to see that in person with him being pushed a little bit more out of his comfort zone to get the full experience from him. I wouldn’t have him do virtual again…it would be taking a spot from a kid who maybe could benefit more from it” (P098).

Difficulties with technological limitations resulted in perceived challenges with virtual care, “especially if there’s a bad WiFi connection, it can be very challenging that way—it makes you feel not as comfortable as it would be having an actual human in front of you” (P136; see also Additional file 1: Table S1, #17). Caregivers also shared discomfort surrounding lack of privacy while participating in virtual sessions: “[youth] don’t always have that private space because virtual a lot of times you have to be home on the computer or somewhere you can sit comfortably and speak openly but if there’s other people around that creates a barrier.” (P087; see also Additional file 1: Table S1, #18) Caregivers also described *difficulty with rapport in virtual care*. They indicated concern that providers could not sense the youth’s needs or read body language. Caregivers stated: “You miss all the facial cues right? It isn’t very good” (P125) and “going into a [video] call with [service] is not as effective as meeting someone face to face, she needs the human connection” (P101; see also Additional file 1: Table S1, #19). Such difficulties with rapport could lead youth to feel as though they were not being supported: “Without seeing someone face to face it’s hard…so my daughter had a hard time understanding or relating to the person so she found it very difficult and ended up in more tears ‘I’m not getting any help’” (P040). Caregivers indicated that providers could not hold youths’ attention the same way virtually:

“Kids are good at multitasking. She can be talking to her therapist while typing on the phone to her friend whereas she wouldn’t have that in a face to face. So I feel that with COVID, not having access to a therapist face to face is huge… [Face to face is] more personable, more in touch with each other.” (P142).

There were also numerous indications of *discomfort with virtual care*, whereby caregivers described apprehension toward their own or their youth’s participation in virtual care: “my husband and I don’t like it at all but [youth] seems to be fine with it” (P125). In some instances, youth did not engage with virtual care, leading caregivers to feel concerned about the appropriateness of virtual care methods to support their youth:

“I wish she was seeing them in person but I understand that it’s not possible…I’ve been there a couple of times and she basically says “I’m okay” and doesn’t get into anything deep when in reality I know she’s struggling with all these different things. But she hasn’t discussed that with her doctor and it makes me frustrated” (P119).

Also permeating descriptions of discomfort with virtual care was *comfort with in-person care*, in that “[virtual care] is not the ideal, obviously, it’s always best person to person” (P047). Participants viewed in-person care as more likely to be beneficial for their youth and preferred in-person care over other alternatives: “[Virtual is] obviously not the best format in which to roll the program out but they had no choice so something was better than nothing…I just think he would’ve gotten more had it been in person” (P098; see also Additional file 1: Table S1, #20–#22).

### Caregiver involvement in help-seeking and access

While caregivers’ involvement in the youth’s care during the pandemic was punctuated by all of the above-described themes, caregivers specifically described additional elements of their *role in help-seeking and access* for the youth. Many caregivers indicated they were better able to help their youth during the pandemic, due to changes in their own personal circumstances. Caregivers also described taking on the role of advocating for their youth’s care, being the “squeaky wheel to get anything progressing” (P125), especially in light of the unique difficulties that arose in accessing MHA services during the pandemic. Conversely, it is important to note that in some cases, caregivers refrained from seeking services during the pandemic, due to perceived unavailability: “I don’t think anybody is seeing people. I haven’t reached out but I know that the way doctor’s offices are now I doubt people are going to have hour long counselling sessions in person…I haven’t looked into it through the pandemic” (P071; see also Additional file 1: Table S1, #23). Caregivers also refrained from seeking services as a means of risk management: “during COVID there’s no way that I would like to have her at a hospital…so I didn’t even call or inquire” (P151). Caregivers also indicated that loss of employment, or the possibility of such, was a significant concern preventing care seeking for the youth. Stressors and financial concerns related to job loss affected caregivers’ ability to support their youth.

“We’ve basically stopped looking for services right now, not even an option basically. We’re just trying to make it day by day. I got laid off from my job and they told me I should be going back [soon] but who knows right now…Anyway financially it’s tough for me right now…So I haven’t looked” (P119).

Caregivers also described their *experiences in help-seeking and access*, expressing great frustration in trying to connect their youth with needed MHA care and support them through their care. One caregiver shared: “It’s been very frustrating, especially during COVID it’s even worse trying to get through to anybody and then also being not being able to go to consults with him.” (P122) Those with prior experience of care expressed concern for caregivers who were new to looking for services and navigating unfamiliar terrain for the first time during the pandemic:

“I feel really bad for people that have situations way worse than us because I think the pandemic has affected kids in a big way and I think people don’t realize how much. On top of that if you are not the type of parent that knows how to reach out and get services or advocate for your kid I think it must be really tough” (P084).

In some instances, caregivers also viewed the increased time together with their youth positively, and highlighted the beneficial aspects this time had on their relationship, with one caregiver expressing: “I was able to reconnect with my daughter” (P045). Despite these positive impacts on youth-caregiver relationships, there was evidence of concern for the youth’s social well-being:

“I spend more physical time with her than ever before so that’s a good thing because I’m not going to work every day, but it still is distressing. I just wish for her to have a friend or somebody she can talk to other than me…if there was somebody that could help in that regard, to check up on her every once in a while” (P119).

Finally, caregivers stressed the importance of remaining flexible and adjusting to the situation the pandemic had presented, noting: “well we have got to learn to live with it, it’s not ending soon” (P125). Caregivers also shared their intentions to continue the journey of supporting their youth: “you gotta do what you gotta do, so, it’s better than not doing” (P136). Caregivers also remained hopeful that despite the pandemic, they would be able to secure appropriate support for their youth: “We haven’t been able to access [service organization] yet because of COVID but I feel hopeful about that. But you know, proof is in the pudding and I’m just kind of waiting and seeing” (P067).

## Discussion

This study explored the impacts of the pandemic for youth with MHA concerns, particularly concerning care access, from the perspectives of caregivers who had taken an active help-seeking role in the youth’s care. Caregivers spoke to changes to everyday life, MHA considerations during the pandemic, availability of and difficulties accessing MHA services, positive and negative aspects of virtual care, and involvement in help-seeking and access for the youth. The youth MHA difficulties and decreased MHA stigma among youth described by caregivers in this study signal the importance and timeliness of engaging with youth in need of care during the pandemic.

Study findings coincided with many others that have been highlighting the impacts of the pandemic on youth MHA; particularly in terms of social isolation, school closures, and disruptions to routine (e.g., [26]). While some caregivers in the current study identified youth MHA improvements as a result of the pandemic and associated restrictions, many emphasized substantial effects of the pandemic in the worsening of their youth’s mental health. However, they also stated the importance of not assuming youths’ MHA concerns were solely attributable to or a direct result of the pandemic, lest this create the presumption that the youth’s MHA needs will improve, unaided, upon return to “normal.” While future implications for youth MHA as the pandemic runs its course are not known, it is critical to treat youths’ immediate MHA needs with due consideration. This is especially imperative as youth often experience dismissive attitudes from adults and providers regarding their MHA concerns, with needs overlooked or their severity not acknowledged [27]. Efforts to prevent negative early experiences with the MHA system can promote ongoing involvement with needed care.

A clear outcome of the pandemic for MHA services has been a shift to virtual care models, with many services rapidly adapting their approaches to be able to serve clients in light of existing protocols and limitations (e.g., [15]). While many participants enthusiastically shared the comfort and conveniences they experienced through virtual care, a finding consistent with other studies conducted during the pandemic [28], in the current study, there was a remarkably pervasive preference for in-person care as well as apprehensions toward virtual care. Attitudes toward virtual care were an important point of discussion around likelihood of participating in virtual care. Accordingly, prior work has highlighted positive youth attitudes toward virtual care as enabling participation, particularly for individual therapy rather than group support [28]. In the current study, this was not limited to the caregivers’ descriptions of the youths’ own attitudes toward virtual care—caregiver attitudes toward virtual care were also described as likely to influence the youth’s future participation in virtual care opportunities. These generally mixed responses regarding virtual care highlight the importance of accounting for youth and caregiver preferences and choice in care, especially early in youth help-seeking where initial care experiences are foundational in building trust with the MHA system and developing positive attitudes toward MHA care in general [29–31]. In particular, findings from the current study suggest that providers should be encouraged to place greater focus on developing rapport over virtual modalities and identify methods that foster youth engagement and participation during virtual sessions.

Participants in the current study had mixed experiences of MHA care accessibility during the pandemic. While it is possible that this was partly a function of timing, in that services closed because they had not yet had a chance to implement safety protocols or virtual supports early on in the pandemic, it is important to consider that these experiences took place in the same jurisdiction over eight months. As such, although some youth did connect with care, these mixed experiences are indicative of an MHA system that did not collectively rapidly respond during the pandemic, leaving a proportion of youth and families unable to access much-needed care. Findings from the current study suggest MHA providers can examine their practices regarding communication with clients, wait list management, supporting clarity regarding resource pathways, and providing clear information to the community regarding program availability. In addition to better supporting access to care, such efforts can greatly mitigate the negative emotional effects—including the frustration, despair, and sense of abandonment by the system—described by caregivers supporting their youth. These experiences also highlight how MHA service access barriers are often oriented around social determinants of health; such barriers existed before the pandemic and appear to have been exacerbated as a result of the COVID-19 pandemic (e.g., [32, 33]). For example, caregivers who shared difficulties accessing care in the current study often described coinciding difficulties related to finances, employment, housing, or transportation, pertaining to themselves and/or their youth. These findings emphasize the need for and importance of policies that direct resources to youth MHA care accessibility in light of the above-mentioned factors, during the pandemic and beyond. Recognizing the important role caregivers can play in youth MHA care access and the burden thus placed on caregivers [34, 35], also indicates a pressing need to ensure the supports available for caregivers of youth with MHA concerns address these factors.

There are a number of limitations to this work worth noting. For one, these interviews were conducted in the context of the healthcare system within a specific jurisdiction of one province of Canada. Therefore, findings may not be generalizable to other geographical locations and healthcare systems. However, these findings regarding the experiences of caregivers of youth with MHA concerns, their perceptions of the impact of the pandemic on their youth’s mental health, as well as their perspectives toward help-seeking during the pandemic may be transferable to other settings and contexts. Secondly, these interviews were conducted with caregivers only, and were drawn from a larger ongoing study that was in place prior to the pandemic and has continued throughout, which does not involve youth participants. Studies qualitatively highlighting youth perspectives are also essential for a more complete picture of the impacts of the COVID-19 pandemic on these youth. Future work may consider caregiver-youth dyads participating in interviews together and separately, to more deeply understand similarities and differences in their perspectives of MHA impacts and care needs during the pandemic and beyond. Finally, all of the caregiver transcripts included in the study were from female-identifying participants. While the purpose of this study was not focused on the female perspective, transcripts from male participants in the parent study did not contain sufficient discussion of the pandemic to be entered into analysis. Focused investigation may be needed to determine whether the themes described in this study are also experienced by male-identifying caregivers in a similar manner.

## Conclusion

Identifying caregiver perspectives of youth MHA needs and service access during the COVID-19 pandemic provided important insights that can help inform approaches to youth MHA care during the pandemic and beyond. Caregivers offer important insights into youth MHA needs [36], particularly during the pandemic [6, 10]. Caregiver perspectives regarding youth MHA needs are also important to understand due to the relationship of youth MHA needs and family functioning with caregiver strain and because of the important role caregivers play in supporting youth access to care [34, 37]. Future work can seek to further understand these impacts from both youth and caregiver perspectives, and should explore models of support that enable equitable access to care that meets youth and caregiver needs and preferences. Understandings of the impacts of disruptions youth and families have experienced during this time in their lives should thus inform ongoing MHA service provision and the implementation of youth and caregiver supports that address these needs.

## Supplementary Information

**Additional file 1: Table S1.** Additional participant quotations.

## Data Availability

The datasets used and/or analysed during the current study are available from the corresponding author on reasonable request.

## References

[1] Children and Youth. Mental Health Commission of Canada. http://www.mentalhealthcommission.ca/English/focus-areas/children-and-youth. Accessed 27 Sep 2016.

[2] Canadian Mental Health Association (2020). COVID-19 effects on the mental health of vulnerable populations.

[3] Nearchou F, Hennessy E, Flinn C, Niland R, Subramaniam SS (2020). Exploring the impact of covid-19 on mental health outcomes in children and adolescents: a systematic review. Int J Environ Res Public Health.

[4] Liang L, Ren H, Cao R, Hu Y, Qin Z, Li C (2020). The Effect of COVID-19 on youth mental health. Psychiatr Q.

[5] Hawke LD, Barbic SP, Voineskos A, Szatmari P, Cleverley K, Hayes E (2020). Impacts of COVID-19 on youth mental health, substance use, and well-being: a rapid survey of clinical and community samples: répercussions de la COVID-19 sur la santé mentale, l’utilisation de substances et le bien-être des adolescents: un sondage rapide. Can J Psychiatry.

[6] Fitzpatrick O, Carson A, Weisz JR (2020). Using mixed methods to identify the primary mental health problems and needs of children, adolescents, and their caregivers during the coronavirus (COVID-19) pandemic. Child Psychiatry Hum Dev.

[7] Nathiya D, Singh P, Suman S, Raj P, Tomar B (2020). Mental health problems and impact on youth minds during the COVID-19 outbreak: cross-sectional (RED-COVID) survey. Soc Health Behav.

[8] Twenge JM, Joiner TE (2020). Mental distress among U.S. adults during the COVID-19 pandemic. J Clin Psychol.

[9] Oosterhoff B, Palmer CA, Wilson J, Shook N (2020). Adolescents’ motivations to engage in social distancing during the COVID-19 pandemic: associations with mental and social health. J Adolesc Health.

[10] Raviv T, Warren CM, Washburn JJ, Kanaley MK, Eihentale L, Goldenthal HJ (2021). Caregiver perceptions of children’s psychological well-being during the COVID-19 pandemic. JAMA Netw Open.

[11] Brannan AM, Heflinger CA, Foster EM (2003). The role of caregiver strain and other family variables in determining children’s use of mental health services. J Emot Behav Disord.

[12] Pfefferbaum B, North CS (2020). Mental health and the Covid-19 pandemic. N Engl J Med.

[13] Liu S, Yang L, Zhang C, Xiang YT, Liu Z, Hu S (2020). Online mental health services in China during the COVID-19 outbreak. Lancet Psychiatry.

[14] Li W, Yang Y, Liu ZH, Zhao YJ, Zhang Q, Zhang L (2020). Progression of mental health services during the COVID-19 outbreak in China. Int J Biol Sci.

[15] Davenport TA, Cheng VWS, Iorfino F, Hamilton B, Castaldi E, Burton A (2020). Flip the clinic: a digital health approach to youth mental health service delivery during the COVID-19 pandemic and beyond. JMIR Ment Health.

[16] Johnson S, Dalton-Locke C, Vera San Juan N, Foye U, Oram S, Papamichail A (2020). Impact on mental health care and on mental health service users of the COVID-19 pandemic: a mixed methods survey of UK mental health care staff. Soc Psychiatry Psychiatr Epidemiol.

[17] Kronenfeld JP, Penedo FJ (2021). Novel Coronavirus (COVID-19): telemedicine and remote care delivery in a time of medical crisis, implementation, and challenges. Transl Behav Med.

[18] Rousseau C, Miconi D (2020). Protecting youth mental health during the COVID-19 pandemic: a challenging engagement and learning process. J Am Acad Child Adolesc Psychiatry.

[19] Tucker JS, D’Amico EJ, Pedersen ER, Garvey R, Rodriguez A, Klein DJ (2020). Behavioral health and service usage during the COVID-19 pandemic among emerging adults currently or recently experiencing homelessness. J Adolesc Health.

[20] Ligus K, Fritzson E, Hennessy EA, Acabchuk RL, Bellizzi K (2021). Disruptions in the management and care of university students with preexisting mental health conditions during the COVID-19 pandemic. Transl Behav Med.

[21] Sandelowski M (2000). Whatever happened to qualitative description?. Res Nurs Health.

[22] Markoulakis R, Levitt A (2020). Considerations in developing a feasibility study of a mixed-methods pragmatic randomized controlled trial of navigation for youth with mental health and/or addiction concerns and their families.

[23] Levinson-King R (2021). Toronto lockdown—one of the world’s longest?.

[24] Braun V, Clarke V (2006). Using thematic analysis in psychology. Qual Res Psychol.

[25] Birks M, Chapman Y, Francis K (2008). Memoing in qualitative research. J Res Nurs.

[26] Marroquín B, Vine V, Morgan R (2020). Mental health during the COVID-19 pandemic: effects of stay-at-home policies, social distancing behavior, and social resources. Psychiatry Res.

[27] Hackett CL, Mulvale G, Miatello A (2018). Co-designing for quality: creating a user-driven tool to improve quality in youth mental health services. Health Expect.

[28] Hawke LD, Sheikhan NY, MacCon K, Henderson J (2021). Going virtual: youth attitudes toward and experiences of virtual mental health and substance use services during the COVID-19 pandemic. BMC Health Serv Res.

[29] Hovish K, Weaver T, Islam Z, Paul M, Singh SP (2012). Transition experiences of mental health service users, parents, and professionals in the United Kingdom: a qualitative study. Psychiatr Rehabil J.

[30] Persson S, Hagquist C, Michelson D (2017). Young voices in mental health care: exploring children’s and adolescents’ service experiences and preferences. Clin Child Psychol Psychiatry.

[31] Rickwood D, Deane F, Wilson C (2007). When and how do young people seek professional help for mental health problems?. Med J Aust.

[32] Bernardini F, Attademo L, Rotter M, Compton MT (2021). Social determinants of mental health as mediators and moderators of the mental health impacts of the COVID-19 pandemic. Psychiatr Serv.

[33] Sareen J, Jagdeo A, Cox BJ, Clara I, Ten Have M, Belik SL (2007). Perceived barriers to mental health service utilization in the United States, Ontario, and the Netherlands. Psychiatr Serv.

[34] Song K, Markoulakis R, Levitt A (2022). Predictors of strain for Canadian caregivers seeking service navigation for their youth with mental health and/or addictions issues. Health Soc Care Community.

[35] McKeague L, Hennessy E, O’Driscoll-Lawrie C, Heary C (2021). Parenting an adolescent who is using a mental health service: a qualitative study on perceptions and management of stigma. J Fam Issues.

[36] Markoulakis R, Chan S, Levitt A (2020). The needs and service preferences of caregivers of youth with mental health and/or addictions concerns. BMC Psychiatry.

[37] Gulliver A, Griffiths KM, Christensen H (2010). Perceived barriers and facilitators to mental health help-seeking in young people: a systematic review. BMC Psychiatry.

