# Supplementary material for: Youth mental health and/or addiction concerns and service needs during the COVID-19 pandemic: a qualitative exploration of caregiver experiences and perspectives
Source: Child Adolesc Psychiatry Ment Health. 2022 May 10;16:35. doi: 10.1186/s13034-022-00471-0 (PMC9088718; doi:10.1186/s13034-022-00471-0)
Supplement: Supplementary file 1 — Additional file 1: Table S1. Additional participant quotations. [file 13034_2022_471_MOESM1_ESM.docx]

Supplement 1

Table S1. Additional Participant Quotations

| Quote # | Quote |
| --- | --- |
| 1 | “It doesn’t help because we’re very much ‘sorry you can’t go out you can’t go and hang at the mall you can’t do any of that’ and I think that stinks for these kids. My daughter keeps saying ‘these are supposed to be the best years of my life in high school and I don’t even get to see my friends’ so that’s sitting on her as well.” (P142) |
| 2 | “She did really well, she did well at school she got a part-time job, she was really doing great. She started socializing more, so we were pretty good…and then when this COVID hit it’s just been so upsetting because she was doing so well and now it’s just some days are good some days are not so good.” (P053) |
| 3 | “She is so anxious and depressed she doesn’t want to leave the house…At least she’s at home I know where she is I’m able to help her if I can and I know she’s not addicted to drugs, I know she’s not drinking.” (P070) |
| 4 | “He wasn’t doing as much cannabis because we were home and so things seemed to calm down. However, because of COVID, there were no demands placed on him where like defiance comes in, like if you can’t go to work you can’t go to school because anyone can’t go to work or go to school, so during COVID it actually it worked to his benefit.” (P097) |
| 5 | “I just went to get some groceries and I wasn’t back for an hour because of the lineup. She was just terrified that something would happen to me and worried that I picked up COVID, this was at the beginning so she was very anxious. And she’s been anxious with me going back to work because I work with children” (P045). |
| 6 | “It was after the pandemic had started I found the psychologist but again there was delay and he didn’t really have space. He put [youth] in but there were whole months when he didn’t have any service.” (P125) |
| 7 | “She was going to some therapy groups and she enjoyed some actually...But they’re all cut off right now because of COVID. She’s alone, she sits by herself all day. It’s really hard because she’s now battling loneliness a lot and just her own thoughts…there’s no outside help other than speaking with a doctor once a week on the phone which usually as soon as she gets on the phone with her it’s like ‘Yeah I’m okay, yes I’m okay’ and then hang up and that’s it. Really she’s not okay, so it’s just frustrating.” (P119) |
| 8 | “I guess with the eating disorders it was the third or fourth person who we finally got hold of… we just weren’t finding anything until eventually we got into the eating disorder clinic but that was months and that was many trips to the hospital and them phoning the eating disorder clinic to try to get our name moved up on the list.” (P114) |
| 9 | “The only contact I got was two weeks into the COVID situation, they let us know that they would be suspending the program at that time. It took a whole four months before they told us ‘sorry he won’t get into the program because we’re not running that program’… I got a phone call maybe a month ago about them planning to restart the program, but I haven’t heard anything else since. So as far as I’m aware they haven’t re-instated the program but I haven’t heard anything else from the psychiatrist to follow up” (P105). |
| 10 | “I was trying to get him to go in [new town] to [AA (Alcoholics Anonymous]) meetings and find a home AA [Alcoholics Anonymous] group and a sponsor…So the meetings are all closed now because of COVID.” (P066) |
| 11 | “If the boy is going to you three times a week to the hospital complaining that he has bugs under his skin wouldn’t you think as a doctor that he would put in psychiatric help, nobody’s getting him any psychiatric help…because I can’t go with him cause of COVID he’s always there on his own. He tells me ‘well they put me on allergy medication for the itch’ and I would tell our family doctor that he thinks he has worms in his body and [the doctor] tells me he’s telling this to the hospital but I don’t know.” (P122) |
| 12 | “The social worker is just trying to slowly develop a rapport with him and earn his trust so right now. He’ll just kind of talk to him about school or about music and stuff. And I think there’s only been like three calls with the social worker, all virtually.” (P137) |
| 13 | “The only positive is the people at her school taking on the responsibility of connecting her with a place or a person that could be most beneficial for her. I’m out of ideas right now…The special education teacher knows my daughter very well and therefore I have placed trust in her to help in making the connection cause she knows [my daughter] so well. So she was able to get us connected to one of the social workers.” (P151) |
| 14 | “The school has actually been our better source of anything…going through the health system we just wait or there’s nothing. At least we’ve got a social worker now who’s trying to weigh in with some opinions and help guide [youth].” (P137) |
| 15 | “I’m quite fine with the phone. My mom’s immunocompromised, she lives with us so we’re all trying to go out as little as possible and be in contact with people as little as possible. So for me I’m like okay this is great I have a little more time, I don’t have to go into a medical building, that’s fine. I would’ve been fine if I had to go too, we’re not so freaked out by COVID that we won’t go out, it’s just the convenience though, if it’s early in the morning you can do it in jammies. Not a bad thing.” (P063) |
| 16 | “Yeah they [youth outreach worker] would come to my house, we didn’t have the same concern, but now I wouldn’t have anybody come into my house. My dad lives with me he has lung cancer, and there’s other medical issues in the house, it’s just not something that we could ever even entertain.” (P071) |
| 17 | “We have other people working from home right now and just generally the neighborhood is a ton of traffic on the internet service. We’ve got the highest internet service that is available and still we have issues almost every single time that we have a virtual session.” (P125) |
| 18 | “Because of COVID-19, the family support meetings, they’re actually doing it videoconferencing now. But because I’m at home and all my kids are here and I have a small house I don’t feel comfortable being at a video conference. My son that’s affected I don’t think he would mind but I don’t want my [other children] just being nosy about it.” (P038) |
| 19 | “There’s a different connection. There’s still a barrier in some way with virtual.” (P118) |
| 20 | “When we’re talking about sensitive subjects I’d rather see that the person’s kind of paying attention to me as opposed to cooking dinner or something while I’m on speaker phone. I’d rather have their undivided attention if I’m going to pour my heart out because I don’t really tell, I keep my situation with my family very private.” (P071) |
| 21 | “I think the group one is better just because it’s more personal, you’re in there with them. Sometimes with online things you tend not to pay attention. I’ve noticed that it’s like there’s a meeting and I’m like lalalala so I just wasn’t comfortable going back. But I would have finished if it was an in person meeting.” (P082) |
| 22 | “In the beginning even with the psychotherapist it was all on Zoom and it was helpful but the last two appointments she was able to physically go and she could not believe the difference in being there physically. It was more beneficial, it was more positive, more productive, all of that.” (P114) |
| 23 | “Not right now there hasn’t been any attempt to try and access. I can imagine that it would be more difficult. When I think about within the facility I work in it’s more virtual appointments or telephone appointments instead of one to one so that can definitely create some angst for people.” (P087) |
